# Giant Spin‐Orbit Torque in Sputter‐Deposited Bi Films

**DOI:** 10.1002/advs.202303831

**Published:** 2023-09-07

**Authors:** Sumin Kim, Hyun‐Woo Lee, Gyung‐Min Choi

**Affiliations:** ^1^ Department of Energy Science Sungkyunkwan University Suwon 16419 South Korea; ^2^ Department of Physics Pohang University of Science and Technology Pohang 37673 South Korea; ^3^ Asia Pacific Center for Theoretical Physics 77 Cheongam‐ro Pohang 37673 South Korea; ^4^ Center for Integrated Nanostructure Physics Institute for Basic Science Sungkyunkwan University Suwon 16419 South Korea

**Keywords:** bismuth, Rashba‐Edelstein effect, spin Hall effect, spin‐orbit torque

## Abstract

Bismuth (Bi) has the strongest spin‐orbit coupling among non‐radioactive elements and is thus a promising material for efficient charge‐to‐spin conversion. However, previous electrical detections have reported controversial results for the conversion efficiency. In this study, an optical detection of a spin‐orbit torque is reported in a Bi/CoFeB bilayer with a polycrystalline texture of (012) and (003). Taking advantage of the optical detection, spin‐orbit torque is accurately separated from the Oersted field and achieves a giant damping‐like torque efficiency of +0.5, verifying efficient charge‐to‐spin conversion. This study also demonstrates a field‐like torque efficiency of −0.1. For the mechanism of the charge‐to‐spin conversion, the bulk spin Hall effect and the interface Rashba‐Edelstein effect are considered.

## Graphical abstract

Spin‐orbit torque from polycrystalline bismuth (Bi) film is investigated using an optical method of magneto‐optical Kerr effect (MOKE). Employing the spatial and vector resolution of the MOKE microscopy, damping‐like torque (*h*_DL_) and field‐like torque (*h*_FL_) are clearly separated from the Oersted field (*h*_Oe_). Large torque efficiencies of +0.5 and −0.1, respectively for *h*_DL_ and *h*_FL_, are achieved.

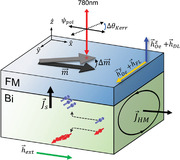

## 1 Introduction

Spintronics aims for electrical control of the spin, and charge‐to‐spin conversion is a key process for both the generation and detection of spin. Especially, efficient charge‐to‐spin conversion is important for low‐power operation of magnetic random access memory. The physical mechanism for charge‐to‐spin conversion can be classified into the bulk and interface effects. The archetypical example of the bulk effect is the spin Hall effect (SHE), which converts a charge current into a transverse spin current inside a bulk material.^[^ ^1^, ^2^, ^3^, ^4^ ^]^ Non‐magnetic heavy metals with strong spin‐orbit coupling (SOC) are known to have a large SHE.^[^ ^5^, ^6^, ^7^ ^]^ The representative example of the interface effect is the Rashba–Edelstein effect (REE), which converts a charge current into a transverse spin polarization at an interface with inversion symmetry breaking.^[^ ^8^, ^9^, ^10^, ^11^, ^12^ ^]^ A similar interface effect occurs at the topological surface states of a topological insulator (TI) through spin‐momentum locking.^[^ ^13^ ^]^

Bismuth (Bi) is the heaviest non‐radioactive element in the periodic table with strong spin‐orbit coupling.^[^ ^14^ ^]^ For this reason, Bi is commonly added to different elements to enhance their conversion efficiency. A large spin Hall angle of 0.24 in Bi‐doped Cu was observed using the inverse spin Hall effect (ISHE) measurement, which converts a spin current into a transverse charge current.^[^ ^15^ ^]^ The spin Hall angle was explained by skew scattering from Bi impurities inside Cu bulk. A giant Rashba spin‐splitting in the band structure was observed at the surface of the Bi/Ag alloy using the angle‐resolved photon‐emission spectroscopy.^[^ ^16^ ^]^ High efficiencies of charge‐to‐spin conversion were also demonstrated via ISHE in Bi/Ag/ferromagnet and Bi/Cu/ferromagnet heterostructures.^[^ ^17^, ^18^ ^]^ In addition, Bi is a host material for TI of, BiSb, Bi_2_Se_3,_ and Bi_x_Se_(1‐x)_, whose surface states were known to have a spin‐momentum locking.^[^ ^19^, ^20^, ^21^ ^]^

Theories predicted a strong spin Hall effect even in a pristine Bi.^[^ ^22^, ^23^ ^]^ However, there is a lack of experimental evidence as to whether Bi itself can be used for charge‐to‐spin conversion. ISHE has been employed in the Bi/ferromagnet bilayer, but the spin Hall angle was rather small with a wide variation from 0.0001 to 0.02.^[^ ^24^, ^25^, ^26^ ^]^ On the contrary, a recent study using 2nd harmonic (2*ω*) Hall measurement reported a gigantic spin Hall angle of >5 of pristine Bi.^[^ ^27^ ^]^ These conflicting results might come not only from the different qualities of Bi films but also from the parasitic effects of electrical measurements.^[^ ^28^ ^]^ In the ISHE experiment employing spin pumping, microwave excitation of ferromagnets may induce not only the spin pumping but also parasitic electric signals from FM via the anisotropic magnetoresistance, the planar Hall effect, and the anomalous Hall effect.^[^ ^29^, ^30^ ^]^ In the 2*ω* Hall experiment, thermoelectric signals often exist, such as anomalous Nernst effect and ordinary Nernst effect.^[^ ^27^ ^]^ Large thermoelectric signals make it difficult to isolate the spin signal.

In this study, to demonstrate an efficient charge‐to‐spin conversion in semimetallic Bi, we optically investigate spin‐orbit torque (SOT) in a Bi/CoFeB heterostructure at room temperature. A charge current in Bi generates a transverse spin current, which induces a torque on the magnetization of CoFeB. SOT is widely used for SHE studies with various heavy metals,^[^ ^5^, ^6^, ^7^, ^10^, ^11^ ^]^ topological insulators,^[^ ^19^, ^20^, ^21^ ^]^ and semiconductors^[^ ^31^, ^32^ ^]^ because the magnetization of ferromagnets is much easier to detect than the spin of non‐magnetic materials. To detect the SOT‐driven change of magnetization, we use the magneto‐optical Kerr effect (MOKE).^[^ ^33^, ^34^ ^]^ Because the detection part (optics) is separated from the generation part (electronics), this MOKE approach can avoid any parasitic effects of the full electrical measurements. From the polarization angle dependence, channel position dependence, and external field dependence, we clearly separate the damping‐like torque (DL), field‐like torque (FL), and Oersted field. We obtain a giant DL efficiency of +0.50 and +0.53, respectively, for the (012) and (003) textured Bi films, which are even larger than those for heavy metals of Pt, Ta, and W.^[^ ^5^, ^6^, ^7^ ^]^ In addition, a significant FL efficiency of −0.13 and −0.09, respectively, for the (012) and (003) textures are obtained. Such a significant FLT cannot be explained by SHE but suggests that the interfacial effect of REE contributes to SOT.

## 2 Results and Discussion

### 2.1 Bi Film Growth and its Properties

**Figure 1:**
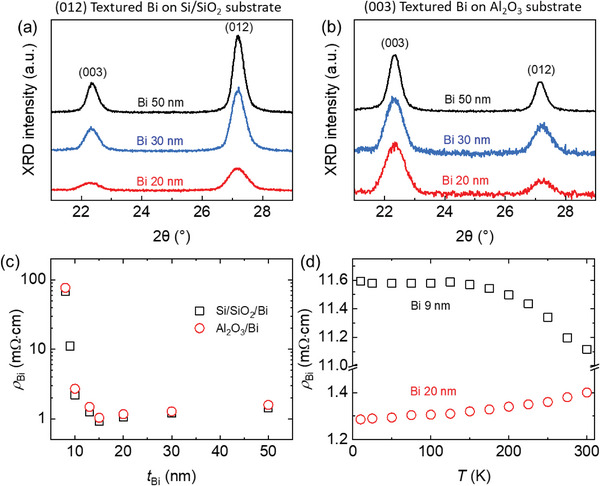
Crystalline texture and electrical resistivity of Bi films. XRD measurement of sputtered Bi films on a) Si/SiO_2_ (300 nm) substrate and b) sapphire(0001) substrate. The XRD peaks at 2*θ* = 22.5° and 2*θ* = 27.2° indicate the out‐of‐plane textures of (003) and (012), respectively. The Si/SiO_2_ substrates promotes the (012)‐dominant texture of Bi, and the sapphire (Al_2_O_3_) substrates promote the (003)‐dominant texture of Bi. The black, blue, and red color indicate the Bi thickness of 50, 30, and 20 nm, respectively. c) The thickness dependence (*t*_Bi_) of electrical resistivity (*ρ*_Bi_) of the Bi films on top of the Si/SiO_2_ substrate (black squares) and sapphire substrate (red circles). d) Temperature (*T*) dependence of *ρ*_Bi_ at with the 9 nm (black squares) and 20 nm (red circles) Bi films on Si/SiO_2_ substrate.

We grow a single layer of Bi film and a bilayer of Bi/CoFeB film using DC sputtering (see Experimental Section). After depositing the metallic films, a 5 nm capping layer of Al_2_O_3_ is deposited using RF sputtering to prevent oxidation of the Bi or CoFeB films. To obtain a different texture, we grow Bi films on top of the Si (001)/SiO_2_ (300 nm) substrates and sapphire (0001) substrates (see Experimental Section). We examine the crystalline structure of Bi films using X‐ray diffraction (XRD). The *θ*−2*θ* scan of the XRD of **Figure** **1**a, shows that Bi films have a polycrystalline rhombohedral structure with a space group of $R\bar{3}m$(no.166). The XRD peaks at 2*θ* = 22.5° and 2*θ* = 27.2° indicate the out‐of‐plane textures of (003) and (012), respectively (Figure 1a,b). (Here, we use the peak notation of hexagonal indices. If we use the rhombohedral indices, the XRD peaks at 2*θ* = 22.5° and 2*θ* = 27.2° correspond to the textures of (111) and (110), respectively.) Importantly, the Si/SiO_2_ and sapphire substrates promote different textures of the Bi films: a (012)‐dominant texture with the Si/SiO_2_ substrates; a (003)‐dominant texture with the sapphire substrates. The intensity ratios between (003) and (012) peaks are 0.3 and 2.0, respectively, with the Si/SiO_2_ and sapphire substrates (Figure 1a,b).

The electrical resistivity of Bi is ≈100 mΩ cm at the Bi thickness of 8 nm, but it reduces to ≈1 mΩ cm at the Bi thickness of 15 nm (Figure 1c). Such a large change in the electrical resistivity is owing to the semimetal‐to‐semiconductor transition of Bi, which is known to occur at a thickness <30 nm.^[^ ^35^ ^]^ To confirm the semimetallic behavior, we measure the temperature dependence of the resistivity (Figure 1d). As the temperature decreases from 300 to 10 K, the resistivity of the Bi 20 nm film shows a decreasing trend, a characteristic of metals, whereas that of the Bi 9 nm films shows an increasing trend, a characteristic of semiconductors. At thicknesses >15 nm, the electrical resistivity remains nearly the same, but the surface roughness increase with the thickness (Note S1, Supporting Information). The root‐mean‐square of a surface's peaks and valleys of the Bi film increases from 3 nm (at the Bi thickness of 15 nm) to 30 nm (at the Bi thickness of 50 nm). To investigate the charge‐to‐spin conversion in the semimetallic phase of Bi with low surface roughness, we choose a Bi thickness range of 10–25 nm. To reduce the Bi roughness even further, a substrate cooling technique can be used during the growth process.^[^ ^28^, ^36^ ^]^

### 2.2 Optical Measurement of Spin‐Orbit Torque

**Figure 2:**
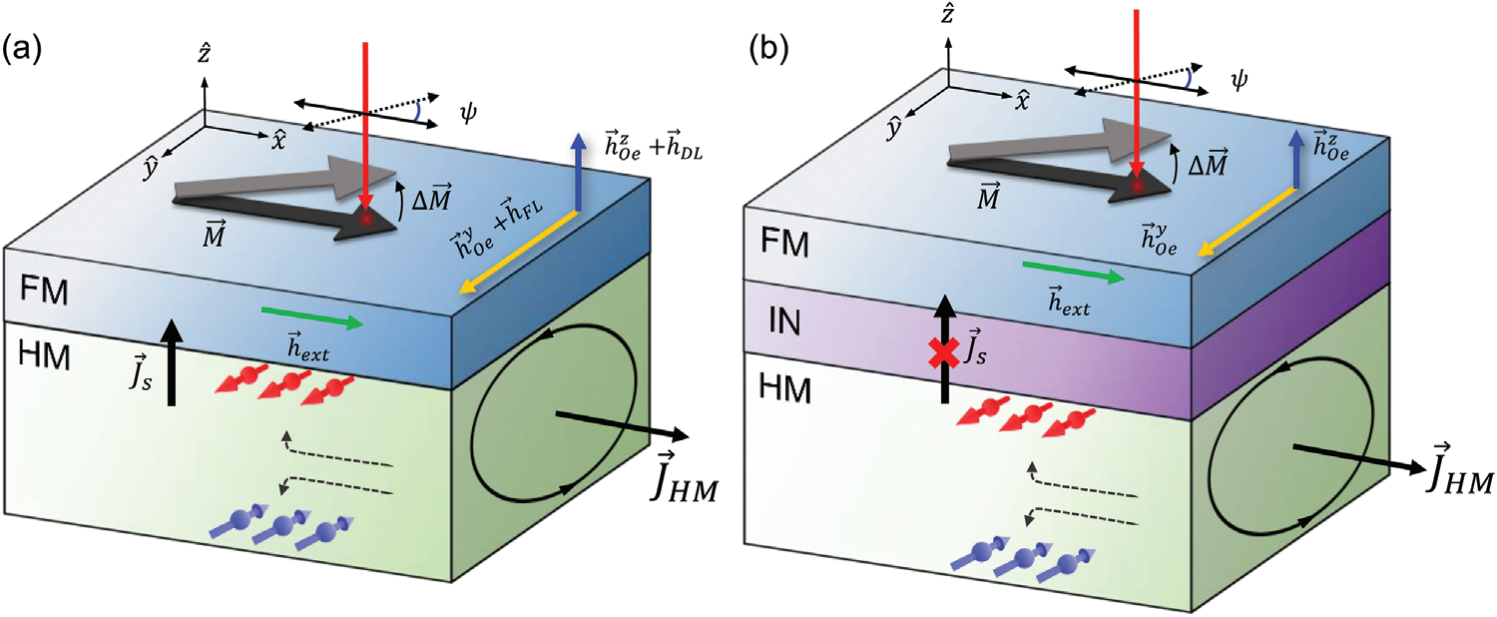
Schematics of the optical spin‐orbit torque measurement. a) When a charge current (*J*) flows in a nonmagnetic heavy metal (HM), Bi or Pt, along the *x*‐direction, a spin current (*J*_s_) flows from HM to a ferromagnetic metal (FM) and induces spin‐orbit torque on magnetization (*M*) of FM. The spin‐orbit torque tilts *M* to *z*‐ and *y*‐directions by damping‐like‐torque field (*h*_DL_) and field‐like‐torque field (*h*_FL_), respectively. The magnetization tilting (Δ*M*) is measured by the polarization (*ψ*) rotation of light. b) With an insulating layer (IN) between HM and FM, a spin current is blocked. Then, only the Oersted field (*h*_Oe_) affects FM without the spin‐orbit torque.

For the SOT measurement, we prepare the HM (20 nm)/FM (5 nm) structure, where HM is a heavy metal of the (012) textured Bi, (003) textured Bi, and (111) textured Pt, and FM is a ferromagnetic metal of Co_40_Fe_40_B_20_. The (111)‐texture Pt is used to confirm the accuracy of our SOT measurement. In addition, as the reference samples, we prepare the HM (20 nm)/IN (5 nm)/CoFeB (5 nm), where IN is an insulating layer of Al_2_O_3_, which blocks the spin current from HM. These multilayer films are fabricated onto a device structure with a channel width of 20 µm using standard photolithography and ion milling. When a charge current (*J*) flows in the HM layer along the *x*‐direction, a generated spin current tilts the magnetization of FM along the *y*‐ or *z‐* direction from the initial *x*‐direction, which is set by an external magnetic field (**Figure** **2**). To enhance the measurement sensitivity, we use a lock‐in technique with a modulation of the charge current (Experiment Section; Note S2, Supporting Information). Then, we measure the current‐induced tilting of magnetization using MOKE (Figure 2). We also confirm that there is no Joule‐heating effect on MOKE (Note S3, Supporting Information). In a polar MOKE geometry (light is incident in a surface normal direction), the Kerr rotation (Δ*θ*_K_) driven by tiling of magnetization can be expressed as.^[^ ^33^, ^34^ ^]^

(1) $$\Delta \theta_{K}\;\left(J\right)=\alpha_{\mathrm{MO}}\Delta m_{z}+\beta_{\mathrm{MO}}\cos\left(2\psi \right)\Delta m_{y}$$

where *α*_MO_ and *β*_MO_ are the linear and quadratic MOKE coefficients of FM, and Δ*m*_z_ and Δ*m*_y_ are the *z*‐ and *y*‐components of the magnetization tilting. Here, Δ*m*_z_ is caused by the damping‐like effective field (*h*_DL_) and the *z*‐component of the Oersted field ($h_{\mathrm{Oe}}^{z}$), and Δ*m*_y_ is caused by the field‐like effective field (*h*_FL_) and *y*‐component of the Oersted field ($h_{\mathrm{Oe}}^{y}$).

**Figure 3:**
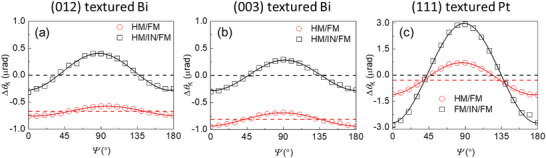
Polarization angle dependence of MOKE. The Kerr rotation (Δ*θ*_K_) of FM in HM/FM bilayer (red circles) and HM/IN/FM trilayer (black squares) with the initial polarization angle (*ψ*) of light from 0 to 180 degrees. With the same FM layer of the CoFeB 5 nm and the IN layer of the Al_2_O_3_ 5 nm, the HM is the a) (012) textured Bi 20 nm, b) the (003) textured Bi 20 nm, and c) the (111) textured Pt 20 nm. The red and black solid lines are the cosine function fittings for the quadratic component of Δ*θ*_K_, driven by Δ*m*_y_. The red and black dashed lines are the offset fitting for the linear component of Δ*θ*_K_, driven by Δ*m*_z_. The zero offset (black dashed line) of the HM/IN/FM structure indicates that the Al_2_O_3_ layer blocks a damping‐like torque. The non‐zero offset (red dashed line) of the HM/FM structure suggests a strong damping‐like torque.

We separate Δ*m*_z_ and Δ*m*_y_ components from the Δ*θ*_K_ dependence on the initial polarization angle (*ψ*) of light (**Figure** **3**). The Δ*m*_z_‐driven Δ*θ*_K_ is responsible for the offset part in Δ*θ*_K_, which does not depend on *ψ*. The Δ*m*_y_‐driven Δ*θ*_K_ is responsible for a cosine dependence on *ψ*. At *ψ* = 45^o^, Δ*θ*_K_ comes from only Δ*m*_z_ without a contribution from Δ*m*_y_. At *ψ* = 0^o^, both Δ*m*_z_ and Δ*m*_y_ contribute to Δ*θ*_K_. In the reference sample of HM/IN/FM, the SOT is blocked by IN, but the Oersted field can penetrate IN and reach FM. When we measure Δ*θ*_K_ of the HM/IN/FM samples at the channel center, where $h_{\mathrm{Oe}}^{z}\;=\;0$ and$\;h_{\mathrm{Oe}}^{y}\;\neq 0$, the offset part in Δ*θ*_K_ becomes zero (Figure 3), indicating that IN blocks a spin current from HM. On the contrary, non‐zero offset part in Δ*θ*_K_ of the HM/FM samples suggests a strong SOT.

### 2.3 Analysis of Damping‐Like Torque

**Figure 4:**
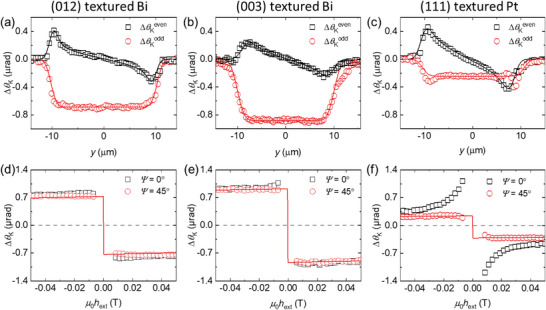
Analysis of damping‐like torque. a–c) The channel position (*y*) dependence of the Δ*m*_z_‐driven Δ*θ*_K_ of the HM/FM bilayer at the initial polarization angle (*ψ*) of light of 45^o^ and the external magnetic field (*h*_ext_) of 50 mT. With the same FM layer of the CoFeB 5 nm, the HM is a,d) the (012) textured Bi 20 nm, b,e) the (003) textured Bi 20 nm, and c,f) the (111) textured Pt 20 nm. Black circles are magnetization tilt due to the z‐component of the Oersted field, obtained by even components under magnetization reversal as {Δ*θ*_K_(+*h*_ext_) + Δ*θ*_K_(−*h*_ext_)}/2. Red squares are magnetization tilt due to the damping‐like torque, obtained by odd components under magnetization reversal as {Δ*θ*_K_(+*h*_ext_) − Δ*θ*_K_(−*h*_ext_)}/2. d‐f) The *h*_ext_ dependence of Δ*θ*_K_ of the HM/HM bilayer at the channel center. The (Δ*m_z_* +Δ*m_y_*)‐driven Δ*θ*_K_ is measured at *ψ* = 0° (black squares) and the Δ*m_z_* driven Δ*θ*_K_ is measured at *ψ* = 45° (red circles). The red line is a fit of the damping‐like torque.

The Δ*m*_z_‐driven Δ*θ*_K_ ($\Delta \theta_{K}^{z}$), measured at *ψ* = 45^o^, has contributions from *h*_DL_ and $h_{\mathrm{Oe}}^{z}$,

(2) $$\Delta \;\theta_{K}^{z}=\alpha_{\mathrm{MO}}\;\frac{h_{\mathrm{DL}}+h_{\mathrm{Oe}}^{z}}{\left|h_{\mathrm{ext}}\right|+h_{\mathrm{dem}}}$$

where *h*_dem_ is the demagnetization field from the saturation magnetization of CoFeB of 1 × 10^6^A m^−1^. We separate *h*_DL_ and $h_{\mathrm{Oe}}^{z}$ from the external field (*h*_ext_) dependence. Whereas $h_{\mathrm{Oe}}^{z}$ only depends on the direction of the charge current and not on the direction of magnetization ($\vec{m}$), which is controlled by ${\vec{h}}_{\mathrm{ext}}$, *h*_DL_ depends on $\vec{m}$ as ${\vec{h}}_{\mathrm{DL}}∥\vec{\sigma }\times \vec{m}$, where $\vec{\sigma }$ is the direction of the spin polarization in HM. Therefore, the even (odd) component of $\Delta \theta_{K}^{z}$ without (with) its sign changing with the sign reversal of *h*_ext_ corresponds to $h_{\mathrm{Oe}}^{z}$ (*h*_DL_). We confirm the clear separation between *h*_DL_ and $h_{\mathrm{Oe}}^{z}$ from the spatial position dependence. $\Delta \theta_{K}^{\mathrm{even}}$ has an antisymmetric profile along the channel position (*y*) because $h_{\mathrm{Oe}}^{z}$ depends on *y* as $h_{\mathrm{Oe}}^{z}\;\left(y\right)=\frac{I_{\mathrm{Bi}}}{2\pi w}\;\ln\frac{w/2+y}{w/2-y}$, where *I*_Bi_ is the current through the Bi layer and *w* is the total width of the channel (**Figure** **4**a–c). However, $\Delta \theta_{K}^{\mathrm{odd}}$ has a flat profile because *h*_DL_ does not depend on the channel position (Figure 4a–c).

We quantify the magnitude of *h*_DL_ by analyzing both $\Delta \theta_{K}^{\mathrm{even}}$ and $\Delta \theta_{K}^{\mathrm{odd}}$. Fitting the antisymmetric profile of $\Delta \theta_{K}^{\mathrm{even}}$ with $\alpha_{\mathrm{MO}}\frac{h_{\mathrm{Oe}}^{z}\left(y\right)}{|h_{\mathrm{ext}}|+M_{s}}$, we determine *α*_MO_. Then, fitting the flat profile of $\Delta \theta_{K}^{\mathrm{odd}}$ with $\alpha_{\mathrm{MO}}\frac{h_{\mathrm{DL}}}{|h_{\mathrm{ext}}|+M_{s}}$, we determine *h*_DL_. When we normalize *h*_DL_ by *J*_Bi_, the current density in the Bi layer, we obtain the *h*_DL_/*J*_Bi_ values of 3.28 and 3.48 × 10^−13^ T m^2^ A^−1^, respectively, with the (012) and (003) textured Bi. *J*_Bi_ is estimated based on the resistivities of the Bi and CoFeB layers (see Experimental Section). Then, we quantify the torque efficiency as *ξ*_DL_ = (2*e*/*ħ*)*M*_s_ *t*_FM_ *h*_DL_/*J*_Bi_, where *t*_FM_ is the thickness of CoFeB. Large *ξ*_DL_ values of +0.50 ± 0.006 and +0.53 ± 0.006, respectively, are obtained with the (012) and (003) textured Bi (Figure 4d,e). Such a large *ξ*_DL_ suggests strong SHE in polycrystalline Bi films, and it is even larger than that of +0.12 ± 0.01 of the (111) textured Pt (Figure 4f). Here, the positive sign indicates that the sign of the spin Hall angle of Bi is the same as that of Pt.

**Figure 5:**
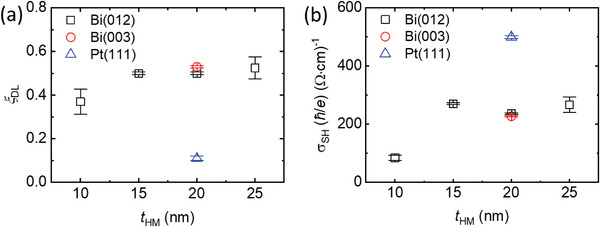
Thickness dependence of damping‐like torque. a) Damping‐like torque efficiency (*ξ*_DL_) and b) spin Hall conductivity (*σ*_SH_) of HM (*t*_HM_)/CoFeB (5 nm) samples. Assuming that spin relaxation length of Bi is much smaller than10 nm, *σ*_SH_ is simply obtained as σ_SH_ = ξ_DL_ /ρ, where *ρ* is the electrical resistivity. Here, HM is the (012) textured Bi (black square), (003) textured Bi (red circles), and (111) textured Pt (blue triangles), with the HM thickness (*t*_HM_) ranging from 10 to 25 nm.

We repeat the same measurement of Figure 4 varying *t*_Bi_ from 10 to 25 nm (Note S4, Supporting Information). *ξ*_DL_ is determined by the spin Hall conductivity (*σ*_SH_) and the Bi thickness (*t*_Bi_) as *ξ*_DL_ = *ρ*_Bi_ *σ*_SH_{1−sech(*t*_Bi_/*l*_s_)}, where *ρ*_Bi_ is the electrical resistivity of Bi and *l*_s_ is the spin diffusion length of Bi. With *t*_Bi_ >15 nm, *ξ*_DL_ of +0.5 is nearly independent of *t*_Bi_, but it reduces to +0.37 at *t*_Bi_ of 10 nm (**Figure** **5**). (With *t*_Bi_ of <10 nm, we were not able to detect the SOT signal because a large resistivity of Bi prevents a current flow.) With conventional heavy metals, such a thickness dependence has been interpreted with *l*_s_ of heavy metals. However, considering that 10 nm is much larger than the typical *l*_s_ of a few nm, we expect that the *ξ*_DL_ reduction at 10 nm is due to the semimetal‐to‐semiconductor transition. The saturated *ξ*_DL_ value of +0.5 leads to *σ*_SH_ of +270 (*ħ*/*e*) (Ω∙cm)^−1^, which is comparable to the theoretical prediction of the intrinsic *σ*_SH_ of +400–1000 (*ħ*/*e*) (Ω∙cm)^−1^.^[^ ^22^, ^23^ ^]^ The spin transparency at the interface is usually less than unity,^[^ ^37^ ^]^ so that the determined values of *ξ*_DL_ and *σ*_SH_ are the lower bound of the true values of Bi. Furthermore, the comparison between the (012) and (003) textured Bi suggests that the anisotropy of SHE is not significant in Bi, which is consistent with the theoretical prediction.^[^ ^23^ ^]^ Considering a close match between the experiment and theories, we conclude that *ξ*_DL_ of the Bi/CoFeB bilayer originates from the intrinsic SHE of Bi.

We note that although *ξ*_DL_ of Bi is about five times larger than that of Pt, *σ*_SH_ of Bi is about two times smaller than that of Pt because of the large electrical resistivity of Bi. In device applications where reducing the transistor size is important and the current supply is the major limitation, a large *ξ*_DL_ is desired. In contrast, in device application where reducing the power consumption is important and the voltage is the major limitation, a large *σ*_SH_ is desired.

### 2.4 Electrical Measurement of Damping‐Like Torque

**Figure 6:**
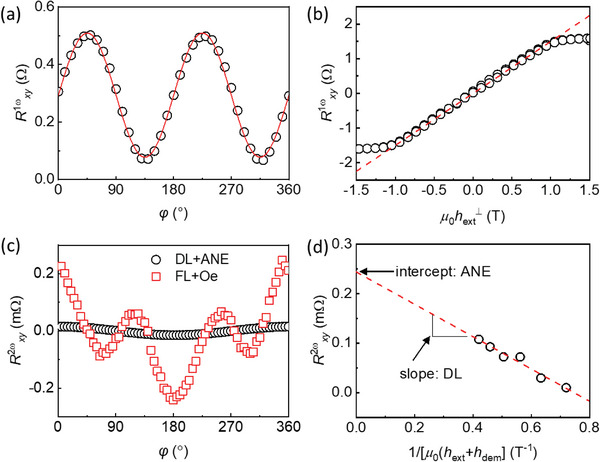
Electrical spin‐orbit torque measurement. a,b) The 1st harmonic Hall resistance ($R_{xy}^{1\omega })$ and c,d) 2nd harmonic Hall resistance ($R_{xy}^{2\omega }$) of the Bi (20 nm)/CoFeB (5 nm) sample. a) The dependence of $R_{xy}^{1\omega }$ on the angle (*φ*) between the magnetic field and charge current with a fixed magnetic field of 0.01 T. The sin 2φ fitting of a) gives the planar Hall resistance, which is used to quantify Δ*m*_y_. b) The dependence of $R_{xy}^{1\omega }$ on the external field along the out‐of‐plane direction ($h_{ext}^{⊥}$). The linear fitting of b) give the anomalous Hall resistance, which is used to quantify Δ*m*_z_. c) The angle (*φ*) dependence of $R_{xy}^{2\omega }$ with a fixed magnetic field of 0.01 T. The cos*φ* term (black circles) corresponds to Δ*m*_z_, driven by damping‐like torque (DL) and anomalous Nernst effect (ANE), and the 2cos^3^ *φ* – cos*φ* term (red squares) corresponds to Δ*m_y_*, driven by field‐like torque (FL) and Oersted field (Oe). d) The external field, along the in‐plane direction, (*h*_ext_) dependence of $R_{xy}^{2\omega }$ at a fixed *φ* of 45^o^. The slope and intercept of d) given DL and ANE contributions, respectively.

To validate our optical measurement, we also perform the conventional electrical measurement of the 2nd harmonic Hall resistance ($R_{xy}^{2\omega }$) with Bi (20 nm)/CoFeB (5 nm) sample (**Figure** **6**; Note S5, Supporting Information). First, we separate the Δ*m*_z_ and Δ*m*_y_ components of $R_{xy}^{2\omega }$ from the angle (*φ*), between the magnetic field and charge current, dependence. Whereas the Δ*m*_y_ component has a 2cos^3^ *φ* – cos*φ* dependence, the Δ*m*_z_ component has a cos*φ* dependence (Figure 6c). Then, out of the Δ*m*_z_ component of $R_{xy}^{2\omega }$, we separate the damping‐like torque (DL) and anomalous Nernst effect (ANE) from the external field (*h*_ext_) dependence. Whereas ANE is independent of *h*_ext_, DL has a 1/(*h*_ext_+*h*_dem_) dependence (Figure 6d). From the field‐dependent part of $R_{xy}^{2\omega }$, we determine the DL efficiency (*ξ*_DL_) of +0.53 ± 0.05, which is consistent with the optical measurement of +0.5 ± 0.006.

### 2.5 Analysis of Field‐Like Torque

To investigate mechanisms other than SHE, we measure FL. According to the theoretical studies, REE at the interface predominantly induces FL on FM.^[^ ^38^, ^39^ ^]^ Experiment studies with a TMD monolayer and InAs quantum well structures demonstrated a significant FL with negligible DL.^[^ ^40^, ^41^ ^]^ To analyze FL, we extract the quadratic (Δ*m*_y_‐driven) Δ*θ*_K_ ($\Delta \theta_{K}^{y}$), taken from the difference of Δ*θ*_K_ at *ψ* of 0^o^ and 45^o^. This $\Delta \theta_{K}^{y}$ has contributions from *h*_FL_ and $h_{\mathrm{Oe}}^{y}$ as

(3) $$\Delta \;\theta_{K}^{y}=\beta_{\mathrm{MO}}\;\frac{h_{\mathrm{FL}}+h_{\mathrm{Oe}}^{y}}{\left|h_{\mathrm{ext}}\right|+h_{\mathrm{ani}}}$$

where *h*_ani_ is the in‐plane anisotropy field of the CoFeB films, which is found to be negligible. Separation of *h*_FL_ and $h_{\mathrm{Oe}}^{y}$ in a single sample is not possible because they have the same symmetry in terms of the external field dependence and spatial position dependence. Instead, we compare $\Delta \theta_{K}^{y}$ in the Bi/CoFeB and Bi/Al_2_O_3_/CoFeB structures. Whereas $h_{\mathrm{Oe}}^{y}$ remains the same in both structures, *h*_FL_ is removed by the Al_2_O_3_ insertion layer. However, caution is required because *β*_MO_ can vary sensitively by the insertion layer.

**Figure 7:**
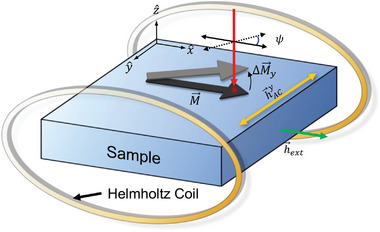
Schematics of magnetic‐field‐driven quadratic MOKE. The Helmholtz coil produces an oscillating (AC) magnetic field (*h*_AC_) along the *y*‐direction, while an electromagnet induces a static (DC) magnetic field (*h*_ext_) along the *x*‐direction. Then, the magnetization (*M*), with the initial direction along the *x*‐direction, oscillates along the *y*‐direction (Δ*m*_y_). The Δ*m*_y_ generates the quadratic MOKE response and enables the determination of the quadratic MOKE coefficient of each sample.

**Figure 8:**
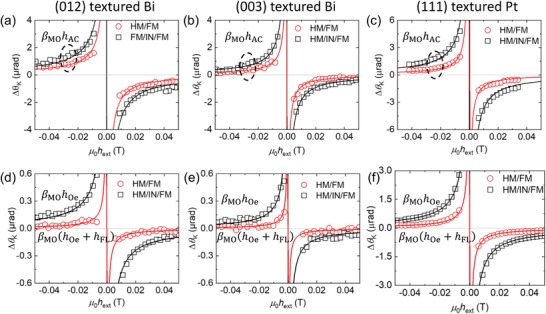
Analysis of field‐like torque. The quadratic (Δ*m*_y_‐driven) Δ*θ*_K_ of FM in the HM/FM bilayers (red circles) and HM/IN/FM trilayer (black squares) driven a‐c) by the AC magnetic field (*h*_AC_) and d–f) by the AC charge‐current (*J*_AC_). With the same FM layer of the CoFeB 5 nm and the IN layer of the Al_2_O_3_ 5 nm, the HM is a,d) the (012) textured Bi 20 nm, b,e) the (003) textured Bi 20 nm, and c,f) the (111) textured Pt 20 nm. The inverse relationship between Δ*θ*_K_ and the external magnetic field (*h*_ext_) indicates that quadratic Δ*θ*_K_ is driven by Δ*m*_y_ without a contribution from Δ*m*_z_. With the same *h*_AC_ in (a‐c), the quadratic Δ*θ*_K_ is determined by the quadratic MOKE coefficient (*β*_MO_). With the same *J*_AC_ in (d–f), the quadratic Δ*θ*_K_ is determined by the *β*_MO_ and competition between the Oersted field (*h*_Oe_) and field‐like torque (*h*_FL_). Especially, the middle IN layer in the HM/IN/FM trilayer blocks *h*_FL_ but does not affect *h*_Oe_.

To determine *β*_MO_ of each sample, we measure the quadratic MOKE with an alternating (AC) magnetic field using a Helmholtz coil (**Figure** **7**; Note S6, Supporting Information). While a static magnetic field from an electromagnet sets an equilibrium magnetization direction to the *x*‐axis, the Helmholtz coil generates the AC magnetic field along the *y*‐direction (in this case, we do not inject charge current so that there is no *h*_FL_ and $h_{\mathrm{Oe}}^{y}$). Then, the field‐driven $\Delta \theta_{K}^{y}$ is expressed as:

(4) $$\Delta \;\theta_{K}^{y}=\beta_{\mathrm{MO}}\;\frac{h_{\mathrm{AC}}^{y}}{\left|h_{\mathrm{ext}}\right|+h_{\mathrm{ani}}}$$

where $h_{\mathrm{AC}}^{y}$ is the AC magnetic field from the Helmholtz coil. Because we applied the same $h_{\mathrm{AC}}^{y}$, the difference in $\Delta \theta_{K}^{y}$ of the Bi/CoFeB and Bi/Al_2_O_3_/CoFeB structures is caused by *β*_MO_. The *β*_MO_ ratios between the Bi/CoFeB and Bi/Al_2_O_3_/CoFeB structures are 0.57 ± 0.06 with the (012) textured Bi and 0.5 ± 0.05 with the (003) textured Bi (**Figure** **8**a–c).

Using the *β*_MO_ ratio, we examine the charge‐current‐driven $\Delta \theta_{K}^{y}$ (Figure 8d–f). $\Delta \theta_{K}^{y}$ of the Bi/CoFeB sample is strongly suppressed compared to that of the Bi/Al_2_O_3_/CoFeB sample, and the $\Delta \theta_{K}^{y}$ ratios are 0.2 ± 0.02 with the (012) textured Bi and 0.17 ± 0.02 with the (003) textured Bi. These values are much smaller than the *β*_MO_ ratio, suggesting that *h*_FL_ strongly cancels $h_{\mathrm{Oe}}^{y}$. By comparing the $\Delta \theta_{K}^{y}$ ratio and the *β*_MO_ ratio of the Bi/CoFeB and Bi/Al_2_O_3_/CoFeB samples, we determine *h*_FL_. The *h*_FL_/*J*_Bi_ values are −8.5 and −6.0 × 10^−14^ T m^2^ A^−1^, respectively, with the (012) and (003) textured Bi. Then, we quantify the FL efficiency as *ξ*_FL_ = (2*e*/*ħ*) *M*_s_ *t*_FM_ *h*_FL_/*J*_Bi_. Large *ξ*_FL_ values of −0.13 ± 0.01 and −0.09 ± 0.01 are obtained, respectively, with the (012) and (003) textured Bi. Here, the minus sign indicates that the direction of *h*_FL_ is opposite to that of $h_{\mathrm{Oe}}^{y}$. Such a large *ξ*_FL_ is comparable to 0.14 of the previous REE system of the MoS_2_/CoFeB structure.^[^ ^40^ ^]^ Interestingly, the *ξ*_FL_ shows a considerable dependence on the crystalline texture of Bi. A further study is required to find out whether the REE mechanism at the Bi/CoFeB interface can explain the magnitude and anisotropy of *ξ*_FL_.

For mechanisms other than SHE and REE, a non‐trivial topology of Bi may contribute to SOT. Unlike the conventional topological insulator, Bi is a second‐order topological insulator, which generates a 1D charge flow along the topological hinge states of Bi.^[^ ^42^ ^]^ To reveal any connection between the hinge states and SOT, further study is required to prove the spin polarization at the hinge states of Bi.

For a comparison between Bi and conventional heavy metals, we also investigate FL in the Pt/CoFeB and Pt/Al_2_O_3_/CoFeB structures. The *β*_MO_ ratio of 0.39 ± 0.03, which is determined from the magnetic‐field‐driven measurement (Figure 8c), is close to the $\Delta \theta_{K}^{y}$ ratio of 0.3 ± 0.03, which is determined from the charge‐current‐driven measurement (Figure 8f). Comparing the *β*_MO_ and $\Delta \theta_{K}^{y}$ ratios, we determine *h*_FL_/*J*_Pt_ of −0.8 × 10^−14^ T m^2^ A^−1^ and *ξ*_FL_ of −0.01, substantially smaller than those with Bi films.

## 3 Summary

To probe the charge‐to‐spin conversion in Bi, we optically investigate SOT in the Bi/CoFeB bilayer. With the crystalline texture of (012) and (003), a polycrystalline Bi film produces giant SOT on CoFeB. The DL efficiency is ≈+0.5 without significant dependence on the crystalline texture. Such a large DL efficiency clearly demonstrates that, in addition to the Bi‐base alloys and compounds, pristine Bi enables a strong charge‐to‐spin conversion via SHE. Furthermore, we observe a significant FL efficiency of ≈−0.1, which cannot be explained by SHE, with a clear dependence on the crystalline texture. The interfacial REE and topological hinge states could be responsible for FL. To understand the exact mechanism, a further study on the correlation among FL, the interface property, and the topology of hinge states would be an interesting topic.

## 4 Experimental Section

### Material Fabrication and Characterization

The Bi single layer, Bi/CoFeB bilayer, and Bi/Al_2_O_3_/CoFeB trilayer were grown using DC (for Bi and CoFeB layers) and RF (for Al_2_O_3_ layer) sputtering at room temperature. The Bi films were sputtered from a single element target, and the CoFeB films were sputtered from a sintered alloy target of Co_40_Fe_40_B_20_ (atomic %). To prevent oxidation, a capping layer of Al_2_O_3_ films was deposited on top of the metallic layers without breaking a vacuum. The processing Ar pressure and base pressure of the sputtering were 3 mTorr and 6 × 10^−7^ Torr, respectively. The electrical resistivities of bare films of Bi and CoFeB were measured using a four‐point‐probe method. The surface morphology of the bare Bi films was measured using a non‐contact mode atomic force microscopy and X‐ray reflectance (Note S1, Supporting Information). The crystalline texture of the bare Bi films was measured using theta/2theta scan of X‐ray diffraction. The magnetic properties of the bare CoFeB films were measured using a vibration sample magnetometer. The bare films of the Bi/CoFeB bilayer and Bi/Al_2_O_3_/CoFeB trilayer to the 20 µm‐width channel devices were patterned using a photolithography and ion milling.

### Magneto‐Optic Kerr Effect Measurement

A linearly polarized laser beam was incident onto the CoFeB layer of the channel devices in a surface normal direction with a center wavelength of 780 nm. After passing through a 20X objective, the beam radius was ≈1.5 µm. The polarization angle of the incident laser beam was controlled by using a motorized rotational stage and a half‐wave plate. When the laser was reflected from the CoFeB layer, its polarization rotated from the initial angle via magneto‐optical Kerr rotation (*θ*_K_). The *θ*_K_ of the reflected laser was measured by a balanced detector (Thorlabs, PDB 450A) with the help of the Wollaston prism.

### Ac Charge‐Current‐Driven Kerr Rotation

The initial direction of the CoFeB magnetization along the *x*‐direction was set by applying a static magnetic field. To induce spin‐orbit torque (SOT) on the CoFeB magnetization, an alternating charge current (*J*_AC_) was applied on the channel devices of Bi/CoFeB and Pt/CoFeB bilayers using an AC current source (Keithly, 6221). The magnitude of *J*_AC_ was 3 and 10 mA, respectively, for the Bi and Pt systems, and the oscillating frequency of *J*_AC_ was 3 kHz. At this frequency range, 1/*f* noise of the laser, which is the major noise source of the measurement system, was minimized. The *J*_AC_ tilts the CoFeB magnetization along the *z*‐ and *y*‐directions by the SOT and the Oersted field. The *J*_AC_‐driven Kerr rotation was collected using a lock‐in technique (Stanford Research Systems, SR830), in which a lock‐in amplifier detects the Kerr rotation at the frequency of *J*_AC_ as Δ*θ*_K_(*J*_AC_) = *θ*_K_(*J*_AC_) – *θ*_K_(*J*_AC_ = 0) (Note S2, Supporting Information).

### Ac Magnetic‐Field‐Driven Kerr Rotation

The initial direction of the CoFeB magnetization along the *x*‐direction was set using a static magnetic field. To tilt the CoFeB magnetization along the *y*‐direction, an alternating magnetic field (*h*_AC_) was applied on the bare films of Bi/CoFeB bilayer and Bi/Al_2_O_3_/CoFeB trilayer using a home‐built Helmholtz coil. The magnitude of *h*_AC_ was 3.5 mT for all systems, and the oscillating frequency of *h*_AC_ was 50 Hz. The frequency was chosen to be low enough to suppress the impedance rise of the Helmholtz coil. The *h*_AC_ tilts the CoFeB magnetization along the *y*‐directions. The *h*_AC_‐driven Kerr rotation was collected using a lock‐in technique, in which a lock‐in amplifier detected the Kerr rotation at the frequency of *h*_AC_ as Δ*θ*_K_(*h*_AC_) = *θ*_K_(*h*_AC_) – *θ*_K_(*h*_AC_ = 0) (Note S6, Supporting Information).

## Conflict of Interest

The authors declare no conflict of interest.

## Supporting information

Supporting Information

## Data Availability

The data that support the findings of this study are available from the corresponding author upon reasonable request.
