# Supplementary material for: Giant Spin‐Orbit Torque in Sputter‐Deposited Bi Films
Source: Adv Sci (Weinh). 2023 Sep 7;10(31):2303831. doi: 10.1002/advs.202303831 (PMC10625106; doi:10.1002/advs.202303831)
Supplement: Supplementary file 1 — Supporting Information [file ADVS-10-2303831-s001.pdf]

ADVANCED SCIENCE Open Access

Supporting Information

for *Adv. Sci.*, DOI 10.1002/advs.202303831

Giant Spin-Orbit Torque in Sputter-Deposited Bi Films

*Sumin Kim, Hyun-Woo Lee and Gyung-Min Choi**

Supplementary Note

Giant spin-orbit torque in sputter-deposited Bi films

Sumin Kim[1], Hyun-Woo Lee[2,3], and Gyung-min Choi [1,4]

[1]Department of Energy Science, Sungkyunkwan University, Suwon 16419, Korea

[2]Department of Physics, Pohang University of Science and Technology, Pohang 37673, Korea

[3]Asia Pacific Center for Theoretical Physics, 77 Cheongam-ro, Pohang 37673, Korea

[4]Center for Integrated Nanostructure Physics, Institute for Basic Science, Sungkyunkwan University, Suwon 16419, Korea.

S1. Surface roughness of Bi films

The morphologies of Bi films are characterized by non-contact mode atomic force microscopy (AFM). AFM results show that the Bi roughness depends on the thickness: the root-mean-square (RMS) of roughness are 4, 10 and 30 nm in 10μm×10μm scan area for 20, 30, and 50nm thickness films, respectively (Fig. S1). In addition, we measured the Bi roughness using X-ray reflectance (XRR) for Bi thicknesses of 15 nm, 20 nm, and 25 nm (Fig. S2). The oscillation of XRR intensity decays rapidly due to the high surface roughness of Bi. The fitting results of the roughness are 5 nm, 4 nm, and 6 nm, respectively, for the Bi thickness of 15, 20, and 25 nm. These values are close to the AFM results of 3, 4, and 8 nm, respectively, for the Bi thickness of 15, 20, and 25 nm. A slight difference between AFM and XRR results can be attributed to the peak height distribution of the surface[1]. When the height distribution of XRR exhibits a non-Gaussian pattern, the reflection coefficient varies. In the case of Bi films, the height distribution has a long tail at higher values.

S2. Charge-current-driven Kerr rotation

To measure SOT, we apply an alternating (AC) charge current onto a channel device of the Bi CoFeB bilayer using an AC current source (Fig. S3). The direction of the AC charge current (J_{AC}) is along the x-direction. The J_{AC}-driven tilting of the CoFeB magnetization is optically measured via magneto-optical Kerr effect (MOKE). The initial polarization of the laser is varied from 0° to 180° from the x-axis using a halfwave plate. At the 0°, both linear and quadratic Kerr rotation is allowed. At 45°, only the linear Kerr rotation is allowed. The Kerr rotation of the reflected laser is measured by a balanced detector with a help of a Wollaston prism. A lock-in amplifier detects the J_{AC}-driven Kerr rotation by collecting the output of the balanced detector at the modulation frequency of the current source. In our setup, the noise in the Kerr rotation detection is dominated by the laser noise. The frequency of the current source is set to 3 kHz, where the spectral noise of the laser is minimized (Fig. S4).

S3. Consideration of thermal effect

To check the thermal effect on the optical measurement, we measured the current dependence of the Kerr rotation for the Bi (10 nm)/CoFeB (5 nm) and Bi (15 nm)/CoFeB (5 nm) samples. The Kerr rotations are linearly proportional to the current density in the Bi layer (Fig. S5). Because the Joule-heating-driven effects, such as anomalous Nernst effect and longitudinal spin Seebeck effect, are proportional to the square of current density, we confirmed

that the thermal effect is negligible in our measurements.

S4. Thickness dependence of damping-like torque

Damping-like torque(DL) of different thicknesses of Bi sample is characterized by the same method of MOKE. The clear anti-symmetric curve of the Oersted field and flat profile of DL are observed for all samples (Fig. S6). Comparison of the anti-symmetry and flat profiles allows a quantifying of the DL efficiency. The DL efficiencies with the Bi thicknesses of 15, 20, and 25 nm are nearly the same. However, the MOKE signal with Bi 25 nm becomes much noisier probably because of an increased roughness. Such a large noise leads to a large error in the DL efficiency. With the Bi 10 nm, the noise in the MOKE signal is similar to those with the Bi 15~20 nm, but the error in the DL efficiency is high owing to a large uncertainty in the electrical resistivity.

To analyze the torque efficiency, one need to know the current density in the Bi layer, magnetization of the CoFeB layer, and magneto-optic constant of the Bi/CoFeB structure. We summarize this information for the (012)-textured Bi, (003)-textured Bi, and (111) textured Pt on the Table S1.

S5. Second harmonic hall measurements

To confirm accuracy of our results, we conducted SOT measurement electrically using second harmonic hall measurements. The hall bar structured Bi (20 nm)/CoFeB(5 nm) device is fabricated on Si/SiO_2 substrate which Bi texture is (012). The sample is mounted on motorized rotation stage and scanned transverse hall voltage 360° range with 5° step. The first harmonic hall resistance($R_{xy}^{1\omega}$) and second harmonic hall resistance($R_{xy}^{2\omega}$) are given by[2]

$$R_{xy}^{1\omega} = R_{\mathrm{AHE}} \cos\varphi - R_{\mathrm{PHE}} \sin^2\varphi \sin(2\varphi), \quad \text{(S1)}$$

$$R_{xy}^{2\omega} = \left[\left(R_{\mathrm{AHE}} \frac{h_{DL}}{h_{ext}} + I_0 \alpha \nabla T\right) \cos\varphi + 2R_{PHE}(2\cos^3\varphi - \cos\varphi) \frac{h_{FL} + h_{Oe}}{h_{ext}}\right], \quad \text{(S2)}$$

where R_{AHE} and R_{PHE} are anomalous hall resistance and planar hall resistance. According to Eq. (S2), the DL and FL part have different dependence of external magnetic field. The cosφ and (2cos$^3\varphi$−cosφ) contributions of $R_{xy}^{2\omega}$ are separated from the fitting of raw data with respect to the angle(φ) between external magnetic field and charge current. The cosφ contribution includes anomalous Nernst effect(ANE) and DL term. To separate DL and ANE, the cosφ contribution of $R_{xy}^{2\omega}$ is measured under different external field (h_{ext}) of 0.2T, 0.4T, 0.6T, 0.8T, and 1T at fixed φ of 45°, where the (2cos$^3\varphi$−cosφ) contribution vanishes. To eliminate offset

component which comes from misalignment, $R_{xy}^{2\omega}$ is also measured with different angles of 0°, 180°, and 325°. The anomalous hall resistance is estimated as 1.58Ω by out-of-plane external magnetic field dependence. From the slope and the intercept of linear fitting of cosφ contribution of $R_{xy}^{2\omega}$ with respect to 1/(h_{ext}+h_{dem}), we obtain damping-like effective field(h_{DL}) of (2.05 ± 0.2)×10^{-4}T and anomalous Nernst effect(R_{ANE}) of 0.244mΩ, respectively. DL efficiency of 0.525±0.05 is consistent with our MOKE measurements.

S6. Magnetic-field-driven Kerr rotation

To measure the quadratic MOKE coefficient of CoFeB, we apply an alternating magnetic field onto bare films, not patterned, of the Bi/CoFeB bilayer and Bi/Al_2O_3/CoFeB trilayer using the Helmholtz coil (Fig. S7). The AC magnetic field (h_{AC}) direction is along the *y*-direction. The h_{AC}-driven tilting of the CoFeB magnetization is optically measured via MOKE. The initial polarization of the laser is fixed to 0°, where the quadratic Kerr rotation is allowed. A lock-in amplifier detects the h_{AC}-driven Kerr rotation by collecting the output of the balanced detector at the modulation frequency of the Helmholtz coil. The frequency of the Helmholtz coil is set to 50 Hz. To avoid the noise from the power supply at 60 Hz, we used a narrow bandwidth setting (equivalent noise bandwidth) of 0.125 Hz. We confirmed that the quadratic MOKE signal is nearly independent of the frequency in the range of 30~50 Hz (Fig. S8). The magnitude of h_{AC} is determined by the current to the Helmholtz coil, which is determined as V/Z_{HC}, where V is the maximum voltage of our current amplifier, and Z_{HC} is the inductance of the Helmholtz coil (Fig. S9). We applied a fixed h_{AC} of 4×10^{-4}T for all samples.

References

[1] V. L. Mironov, O. G. Udalov, B. A. Gribkov, A. A. Fraerman, *J. Appl. Phys.* **2008**, 104, 064301

[2] C. O. Avci, K. Garello, M. Gabureac, A. Ghosh, A. Fuhrer, S. F. Alvarado, P. Gambardella, *Phys. Rev. B* **2014**, 90, 224427

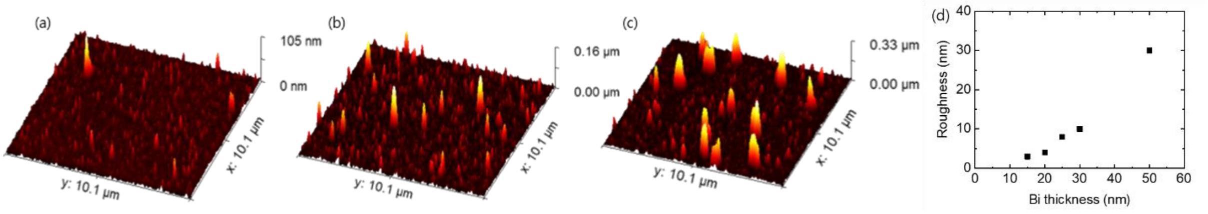

Fig. S1. Morphologies of Bi films of (a) 20, (b) 30, and (c) 50 nm. Bi films are characterized by non-contact mode atomic force microscopy. The root-mean-square of roughness is 4, 10, and 30 nm, respectively. (d) Summary of Bi roughness in the thickness range of 15~50 nm.

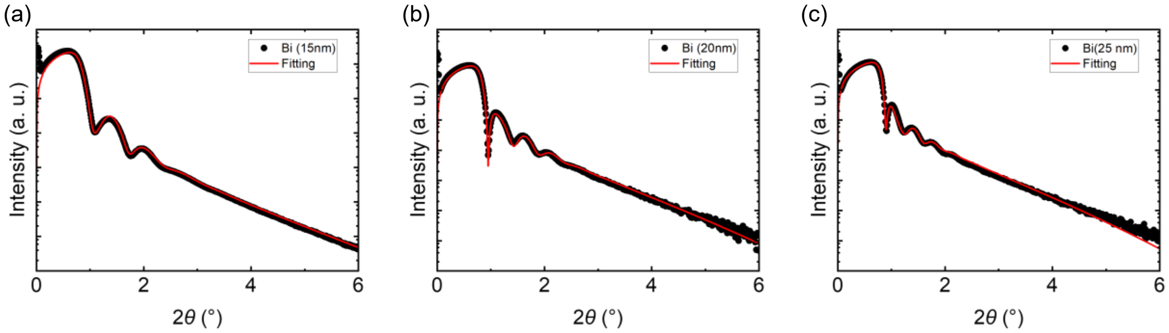

Fig. S2. X-ray reflectometry of Bi films of (a) 15 nm, (b) 20 nm, and (c) 25 nm. Rapid decay of oscillation observed due to the high surface roughness. Fitting of decaying leads to the roughness of 5, 4, and 6 nm, respectively, for the Bi thickness of 15, 20, and 25 nm.

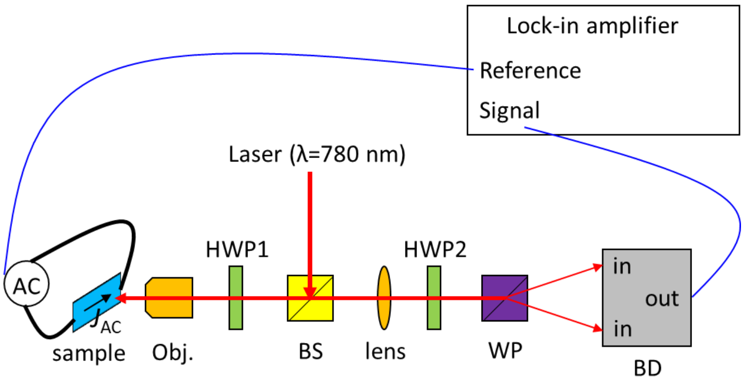

Fig. S3. Experimental setup for the charge-current-driven Kerr rotation. An alternating (AC) charge current (J_{AC}) is applied to the sample along the *x*-direction using an AC current source. The spin-orbit torque tilts the magnetization from the initial *x*-direction, which is set by a static magnetic field. The magnetization tilting is measured optically. A laser with a wavelength of 780 nm is focused on the sample using an objective lens (obj.), and its initial polarization is varied from 0° to 180° by a halfwave plate (HWP1). A reflected laser is collected by a beam splitter (BS), focusing lens, halfwave plate (HWP2), Wollaston prism (WP), and balanced detector (BD). The HWP2 is used to rotate the polarization of the laser to 45°, a balancing point for BD. The WP splits the *x*- and *y*-polarized laser. The BD measured the intensity difference between the *x*- and *y*-polarized laser. The output of BD is connected to the signal input of a lock-in amplifier. The lock-in amplifier collects the Kerr rotation at the modulation frequency of the AC current source.

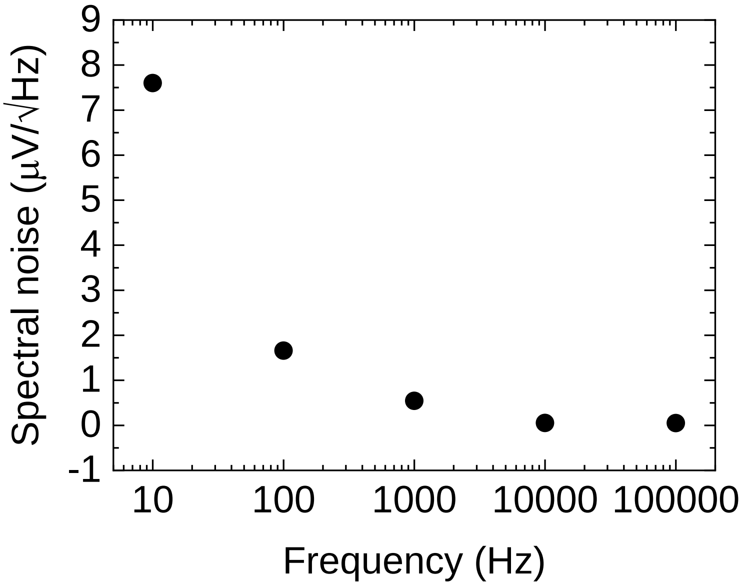

Fig. S4. Spectral noise of laser. The laser noise is measured by injecting a laser directly into the photodetector without a sample or current source. A lock-in amplifier measures the spectral noise of the laser at the reference frequencies from 10 to 100 kHz. The spectral noise decreases with frequency, and it saturates to ~0.1 μV/√Hz at frequencies over 3 kHz.

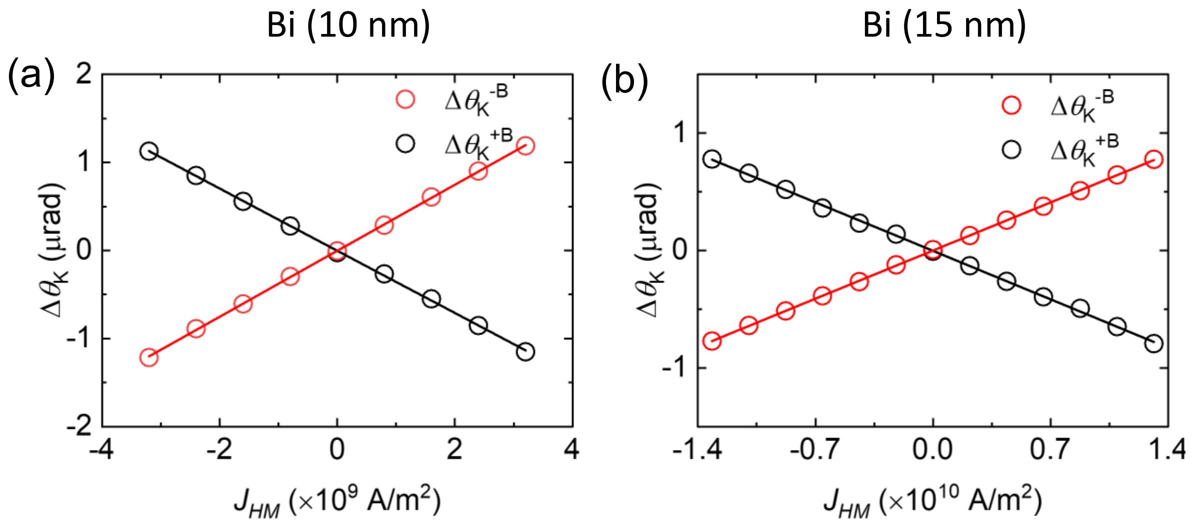

Fig. S5. Current density dependence of Kerr rotation. The current-driven Kerr rotation in (a) Bi (10 nm)/CoFeB (5 nm) and (b) Bi (15 nm)/CoFeB (5 nm) samples. Red and black circles are representing Kerr rotation under opposite external magnetic fields. Kerr rotations are linearly proportional to current density in the Bi layer(J_{HM}). Thermal signal, which is proportional to the square of J_{HM}, is negligible in the MOKE signal.

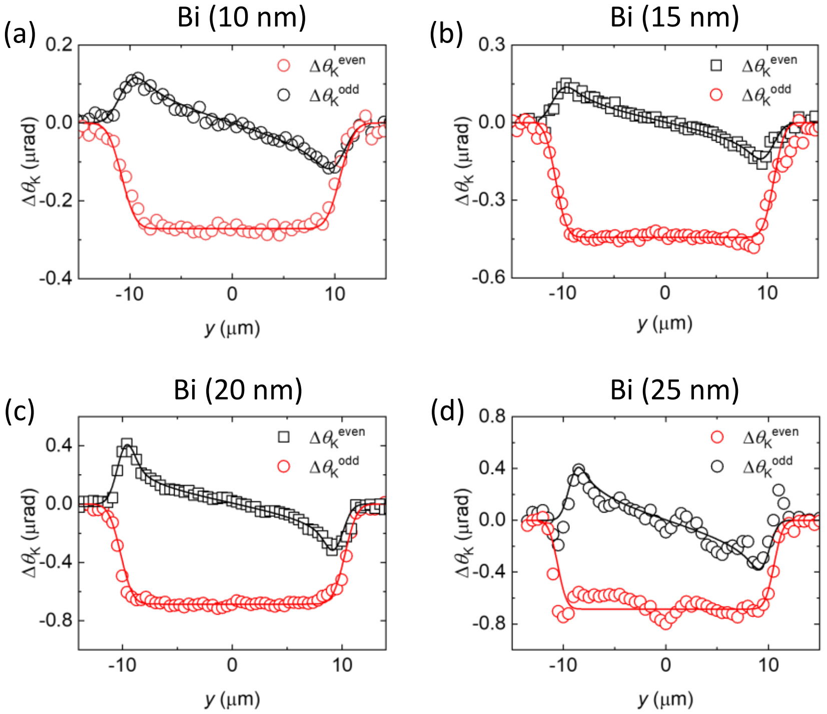

Fig. S6. Bi thickness effect of SOT measurement. (a)~(d) The channel position (y) dependence of the Δm_z-driven $\Delta\theta_K$ of the Bi/CoFeB bilayer at the initial polarization angle (ψ) of light of 45° and the external magnetic field (h_{ext}) of 50 mT. With a fixed CoFeB thickness of 5 nm, the Bi thickness is (a) 10 nm, (b) 15 nm, (c) 20 nm, and (d) 25 nm. The damping-like torque efficiency, determined from the ratio between the even ($\boldsymbol{\Delta\theta_K^{even}}$) and odd ($\boldsymbol{\Delta\theta_K^{odd}}$) parts of the Kerr rotation, is nearly independent of the Bi thickness after 15 nm. However, the $\Delta\theta_K$ signal with the Bi 25 nm gets much noisier compared to those with smaller Bi thicknesses.

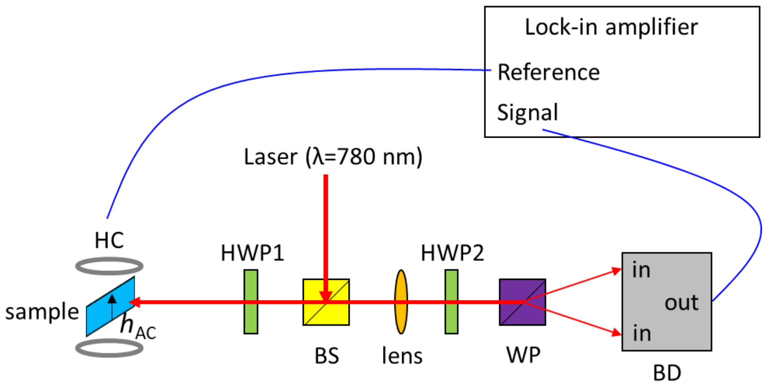

Fig. S7. Experimental setup for the magnetic-field-driven Kerr rotation. An alternating (AC) magnetic field (h_{AC}) is applied to the sample along the y-direction using the Helmholtz coil (HC). The h_{AC} tilts the magnetization from the initial x-direction, which is set by a static magnetic field. The magnetization tilting is measured optically. Here, an objective lens is removed because a long-ranged h_{AC} can induce the Faraday rotation on the objective lens. The initial polarization of the laser is fixed to 0° by a halfwave plate (HWP1). A reflected laser is collected by a beam splitter (BS), focusing lens, halfwave plate (HWP2), Wollaston prism (WP), and balanced detector (BD). The output of BD is connected to the signal input of a lock-in amplifier. The lock-in amplifier collects the Kerr rotation at the modulation frequency of the Helmholtz coil.

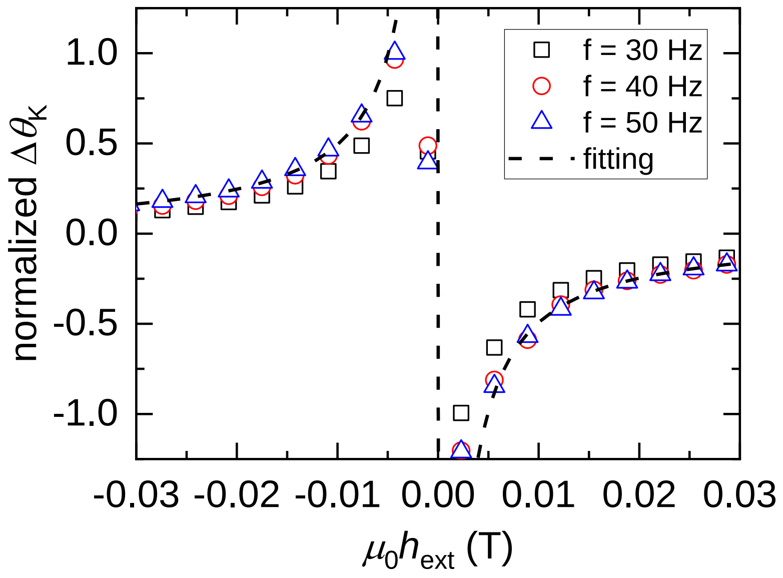

Fig. S8. Modulation frequency effect on the quadratic MOKE measurement. The quadratic MOKE measurements of a Co 30 nm films with modulation frequencies of 30 Hz (black squares), 40 Hz (red circles), and 50 Hz (blue triangles) for the Helmholtz coil. A black dashed line indicates that the quadratic MOKE, arising from ΔM_y, is inversely proportional to the external field (h_{ext}).

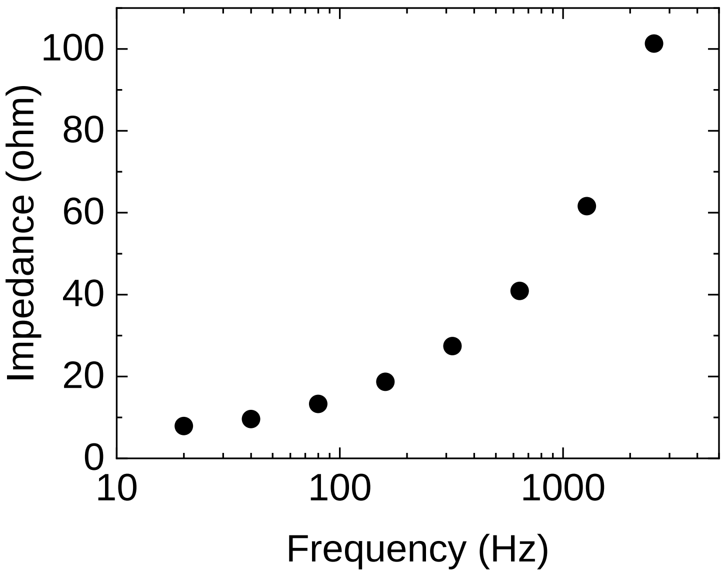

Fig. S9. Impedance of the Helmholtz coil. With applying an oscillating current, the voltage drop of the Helmholtz coil is measured. The impedance is determined as the ratio between the voltage drop and current. As the frequency of the current increase, the impedance of the Helmholtz coil increases from 7.9 Ohm at 0 Hz (static mode) to 101 Ohm at 2.56 kHz.

Parameter	Bi(012)	Bi(003)	Pt(111)
ρ_{HM} ($\mu\Omega \cdot m$)	10.5	11.5	1.1
I_{HM} (mA)	2.076	2.012	9.9
J_{HM} (10^9 A/m^2)	5.19	5.03	24.75
M_s (T)	1.26	1.26	1.37
α_{MO} (μrad)	-5300±100	-6750±50	-2120±30
β_{MO} (μrad)	-42±0.4	-20±0.8	-21±0.1
h_{DL}/J_{HM} (10^{-14}T·m^2/A)	3.28±0.04	3.48±0.04	0.67±0.012
h_{FL}/J_{HM} (10^{-15}T·m^2/A)	-8.48±0.54	-6.0±0.66	-0.75±0.08
ξ_{DL}	0.50±0.006	0.53±0.006	0.11±0.01
ξ_{FL}	-0.126±0.01	-0.09±0.011	-0.012±0.0013

Table S1. Parameters for the spin-orbit torque analysis of the (012) textured Bi (20 nm)/CoFeB (5 nm), the (003) textured Bi (20 nm)/CoFeB (5 nm), and the (111) textured Pt (20 nm)/CoFeB (5 nm) samples.
